# An implementation history of primary health care transformation: Alberta’s primary care networks and the people, time and culture of change

**DOI:** 10.1186/s12875-020-01330-7

**Published:** 2020-12-05

**Authors:** Myles Leslie, Akram Khayatzadeh-Mahani, Judy Birdsell, P. G. Forest, Rita Henderson, Robin Patricia Gray, Kyleigh Schraeder, Judy Seidel, Jennifer Zwicker, Lee A. Green

**Affiliations:** 1 School of Public Policy / Department of Community Health Sciences, Cumming School of Medicine, University of Calgary, DTC547 – 906 8th Avenue SW, Calgary, AB T2P 1H9 Canada; 2 Saskatchewan Population Health and Evaluation Research Unit, University of Regina, Regina, Canada; 3 IMAGINE Citizens Collaborating for Health, Calgary, Canada; 4 Department of Family Medicine, Cumming School of Medicine, University of Calgary, Calgary, Canada; 5 Department of Pediatrics, Cumming School of Medicine, University of Calgary, Calgary, Canada; 6 Department of Community Health Science, Cumming School of Medicine, University of Calgary, Alberta Health Services, Calgary, Canada; 7 School of Public Policy / Faculty of Kinesiology, University of Calgary, Calgary, Canada; 8 Department of Family Medicine, Faculty of Medicine and Dentistry, University of Alberta, Edmonton, Canada

**Keywords:** Primary care, Policy, Fee for service, Quality improvement, Patient medical home

## Abstract

**Background:**

Primary care, and its transformation into Primary Health Care (PHC), has become an area of intense policy interest around the world. As part of this trend Alberta, Canada, has implemented Primary Care Networks (PCNs). These are decentralized organizations, mandated with supporting the delivery of PHC, funded through capitation, and operating as partnerships between the province’s healthcare administration system and family physicians. This paper provides an implementation history of the PCNs, giving a detailed account of how **people**, **time**, and **culture** have interacted to implement bottom up, incremental change in a predominantly Fee-For-Service (FFS) environment.

**Methods:**

Our implementation history is built out of an analysis of policy documents and qualitative interviews. We conducted an interpretive analysis of relevant policy documents (*n* = 20) published since the first PCN was established. We then grounded 12 semi-structured interviews in that initial policy analysis. These interviews explored 11 key stakeholders’ perceptions of PHC transformation in Alberta generally, and the formation and evolution of the PCNs specifically. The data from the policy review and the interviews were coded inductively, with participants checking our emerging analyses.

**Results:**

Over time, the PCNs have shifted from an initial Frontier Era that emphasized local solutions to local problems and featured few rules, to a present Era of Accountability that features central demands for standardized measures, governance, and co-planning with other elements of the health system. Across both eras, the PCNs have been first and foremost instruments and supporters of family physician authority and autonomy. A core group of **people** emerged to create the PCNs and, over **time,** to develop a long-term Quality Improvement (QI) vision and governance plan for them as organizations. The continuing willingness of both these groups to work at understanding and aligning one another’s **cultures** to achieve the transformation towards PHC has been central to the PCNs’ survival and success.

**Conclusions:**

Generalizable lessons from the implementation history of this emerging policy experiment include: The need for flexibility within a broad commitment to improving quality. The importance of time for individuals and organizations to learn about: quality improvement; one another’s cultures; and how best to support the transformation of a system while delivering care locally.

**Supplementary Information:**

The online version contains supplementary material available at 10.1186/s12875-020-01330-7.

## Background

The transformation of primary care has been identified as central to achieving the quadruple aim of better care; better health outcomes; better provider experiences; and better value for money [1, 2]. Transformation here can be understood in a number of ways, but key among them is the idea that primary care practice becomes less focused on diagnosing and curing disease and more focused on supporting health, wellness, and the effective management of chronic conditions [3, 4]. This can be summed up as a shift from *primary care* to *primary health care* (PHC), with a resulting emphasis on population health, prevention, and service delivery integration [3, 5, 6] that promises to bend the cost curve as it moves care upstream from hospitals [7, 8].

In this research we follow Hutchison et al*’s* [9] definition of Primary Health Care (PHC) as a term inclusive of not only Starfield’s [10] principles of primary care, but an even broader spectrum of activities that include population-based approaches to prevention and health promotion. In addition to providing continuous person-focused care for common health problems, first contact access for new health problems, and care coordinated across specialty, acute, and community services, PHC involves a broad array of medical and non-medical services addressing the social determinants of health [11]. There has been intense policy interest in the reform [12], renewal [13], or more latterly transformation [14] towards PHC not just in Canada [2, 15, 16] but around the world [17–22]. Among other positive effects, recent studies have linked reductions in Emergency Department utilization and hospital admissions to PHC-focused state-level investments in US primary care [23, 24].

What follows is an account of incremental and still emergent PHC transformation led by, rather than imposed upon, independent contractor family physicians and supported by system administrators. It takes place in Alberta, one of Canada’s 14 health jurisdictions (1 federal, 10 provincial and 3 territorial ministries of health), each of which is responsible for the governance, organization, and delivery of health services to specific populations. We present an implementation history [25] describing the ecology and evolution of particular transformation efforts undertaken not in an integrated, centrally managed primary care system, but rather in a single payer, fee-for-service (FFS) environment. In this sense Alberta becomes a case study that focuses on understanding the productive possibilities that exist in the space between PHC aims as written and changes in practice on the ground. We are not presenting an account of PHC fully realized, but rather of the flexible on-the-ground interpretations and prioritizations of those seeking to move towards PHC as a broader idea. As such, we co-create, along with our participants, an oral history of what actually happened as a series of PHC transformation policies shaped action. Drawing on policy analysis and key stakeholder interviews we present an implementation history that describes the interactions between people, time, and culture that have supported the bottom up emergence and evolution of Alberta’s PHC-focused Primary Care Networks (PCNs).

To understand this bottom up phenomenon it is important to understand some key top down policy activities that set the stage for the PCNs to emerge. Canadian efforts to meet what are now seen as the core concerns of the quadruple aim, as well as satisfy local political and clinical labor supply imperatives, have led to near constant changes in Canada’s broader healthcare systems over the preceding decades. However, until the beginning of the new millennium, the organization and funding of primary care with an eye on transforming it into PHC specifically, was not a priority. In 2000, the Canadian federal government created an $800-million Primary Health Care Transition Fund to support provinces and territories in their transformation and redesign efforts [9]. The fund was launched at a time when, just as elsewhere in the G20, healthcare had become a remarkably large portion of national spending [26, 27]. At the time, the fund’s designers set out the following five PHC objectives for the provinces and territories: 1) increasing access across the population to PHC organizations and services, 2) shifting emphasis towards health promotion, chronic disease management, as well as disease and injury prevention, 3) expanding 24/7 access to essential services, 4) establishing inter-disciplinary teams of health care providers, and 5) facilitating integration and co-ordination with other health and social services (institutions and in communities) [28]. The fund’s objectives, then, are the first of a series of efforts to interpret and prioritize specific elements of the broader PHC agenda.

Spurred by the fund, PHC-targeted transformation has become a major area of policy activity in Canada, producing a variety of programs and solutions across the country [16, 29]. Within this wide range of policy innovations, the nature and extent of progress have been variable across jurisdictions, and with limited exceptions, [29–31] provincial and territorial experiences have gone un-shared [32]. This is to say, with PHC transformation efforts taking place in Canada, and thus “outside the confines of centrally managed integrated health systems,” [23] little attention has been paid to the successes and challenges encountered by those implementing policy. With this lost opportunity for mutual learning in mind, this paper tracks the origins and evolution of Alberta’s PCNs as mechanisms for evolving toward PHC. At a time when the concept of PCNs is gaining traction both within Canada [30, 33], and worldwide [34], we describe the specific details of Alberta’s organizations as vectors for the transformation of primary care that have matured over the last 15 years. While there has been significant scholarly and policy interest in the PHC transformation efforts of other provinces – see for example [2, 29, 33, 35, 36] – Alberta’s PCNs have received little focused attention in the literature. Having achieved not just significant longevity and maturity, but buy-in from the province’s family physicians even as greater accountability and control have been introduced into their FFS operations, this implementation history of the PCNs present a compelling case study of bottom up, incremental PHC transformation in action.

### The form and context of the PCNs

Alberta, a landlocked western province in the Canadian federation, is the nation’s fourth most populous jurisdiction and home to approximately 4.1 million residents spread over 640,000 km^2^. This population is concentrated in two major cities: Calgary (1.285 million) and Edmonton (972,000). As elsewhere in the federation the province of Alberta, aligning itself with the Canada Health Act, seeks “to facilitate reasonable access to health services without financial or other barriers” [26] to Albertans, and is responsible for the delivery of care. Alberta’s particular policy approaches to delivering care have, arguably, been subject to one of the most radical administrative shifts in Canada: in 2008, nine regional health authorities were centralized into the province’s largest single employer, Alberta Health Services (AHS) [37]. With 110,000 staff, but a very limited role in the delivery of primary care, AHS’ central focus is the province’s acute care system. It is Alberta Health – the provincial Ministry of Health (MoH) – which has tended to engage with, and has always paid, family physicians on an almost exclusively FFS basis [38]. Fee schedules are determined through negotiations between the Alberta Medical Association (AMA) and the MoH [39], The potential for the expansion of highly successfully alternative payment models [40] as well as the outright elimination of FFS have been introduced by the most recent provincial budget [41] but, for the moment, it remains Alberta’s foundational approach to funding primary care and its ongoing transformation towards PHC.

In 2003, drawing on the federal Primary Health Care Transition Fund mandate, the PCNs were created through an agreement between the AMA, the MoH, and the regional health authorities that would eventually be merged into AHS. The three parties signed an 8-year (2003–2011) Trilateral Master Agreement to support Local Primary Care Initiatives (LPCIs). The initial relationship between the AMA, the MoH, and AHS was one of equals that required consensus on the form of, and priorities for, what would come to be called the PCNs. That consensus tended, in the early years, to be driven by the grassroots concerns of family physicians working through the AMA. However, between 2008 and 2011, the MoH became pre-eminent and began exerting, in concert with AHS, greater central control over the PCNs [37, 42, 43].

Alberta’s PCNs are positioned between the MoH and frontline family physicians. More often than not, they operate at the supra-clinic level, joining together practices with one or more physicians. Membership is voluntary, with approximately 84% of Alberta’s family physicians choosing to sign a contract with a PCN [44]. Each PCN is established through a joint venture agreement between AHS and a group of family physicians who have formed themselves into a non-profit corporation. This joint venture is devoted to collaboratively identifying local priorities and developing programs and services. The PCN that emerges from the joint venture is accountable to the MoH under the terms of a three-year grant agreement that flows money from the ministry according to a visit-based algorithm that attributes patients to physician members of the originating non-profit corporation. In 2019, those grant agreements were valued at C$62 per patient per year. PCNs direct this funding towards: 1) non-physician health professionals and administrative staffing, 2) chronic disease management, 3) providing 24/7 access to care, 4) expanded office hours, and 5) premises and equipment. In this way the PCNs use pooled resources – the capitation funds – to cover their own administrative costs and provide a range of services to their members. In turn, member physicians continue to bill the MoH on a FFS basis for the delivery of primary care services; with each member’s capitation grant being directed entirely towards the PCN and its locally adapted basket of PHC support services. If a primary care physician declines to become a member of a PCN, they will receive neither the capitation grant, nor any of the services offered to PCN members.

**Fig. 1:**
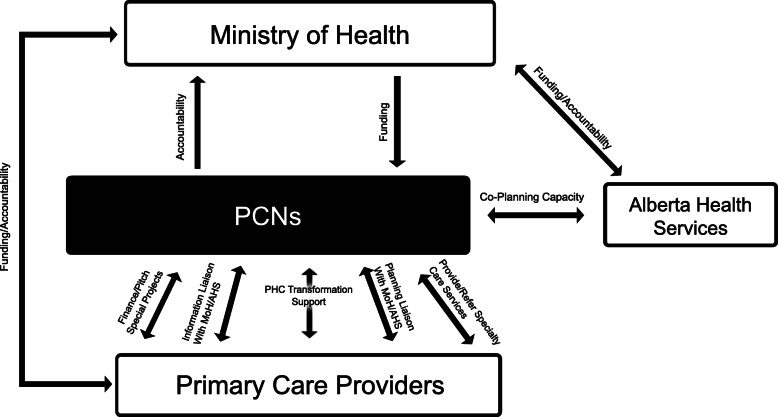
PCNs' relative position in Alberta's healthcare system

From their position as an intermediate layer in Alberta’s healthcare policy eco-system, the PCNs operate in three directions. Looking upwards from their perspective, they deliver funding from, and extract accountability for, the MoH. Looking laterally towards their co-venture partners, they provide a point of contact with, and co-planning capacity to, AHS, the manager and operator of the broader system. And finally, looking downwards to their membership, they provide financial, administrative, technical, planning and PHC-transformation-management support to individual primary care providers (Fig. 1).

## Methods

We conducted an interpretive qualitative study to better understand the PCNs’ implementation history, and so generate a rich, contextualized account of these particular PHC transformation efforts. We approach the ecology of the PCNs as being a recursive interaction between policy documents, human experiences, and institutional history [25]. In taking this approach we developed a recursive two phase method aimed at highlighting the factors – what would become in our findings the people, time, and culture – which have interacted to see the PCNs not just survive, but incrementally evolve from the bottom up in a FFS environment. In the initial documentary phase, we conducted a background policy analysis and in the subsequent interview phase, we focused on stakeholder perspectives on and experiences of those policies.

We analyzed a broad range of policy documents published since the 2005 launch of the first PCN. From an initial list of relevant policies and websites developed by the research team and co-authors on the paper, we read and either snowball sampled, or were directed towards further documents by our key informant interview participants. The central documents in our analysis were: 1) Auditor General of Alberta reports since 2012 (when PCNs first appeared in these reports) up to 2018 (time of conducting the research) (*n* = 7), with a focus on the Health section; 2) Primary Care Policy Manuals as published by the MoH (*n* = 2); 3) Report Card: Status of the Trilateral Master Agreement (2005); 4) Alberta Health Services’ (AHS) Health Plan (2010–2015): Improving Health for All Albertans (2010); 5) The Ministry of Health’s (MoH) Primary Health Care Evaluation Framework (2013); 6) Alberta Primary Health Care Strategy (2014); 7) Primary Care Network Review (2016) and the Primary Care Initiative Evaluation (2011); 8) the AMA’s PCN Evolution Vision and Framework (2013);, 9) 2018–19 Alberta Medical Association (AMA) business plan; 10) sample business plans for PCNs (*n* = 2), and; 11) the websites of the MoH, AHS, AMA, Health Quality Council of Alberta, the College of Physicians and Surgeons of Alberta, and the College of Family Physicians of Canada. Indeed, much of the background presented above relies heavily on readings of these and a broader list of policy documents, cross checked first in the course of the interviews, then again in participant checks of emerging findings, and finally in the preparation of this manuscript. Beyond providing background, our documentary analysis specifically helped us identify stakeholders engaged with PHC transformation, as well as the content of proposed policies and strategies; the evidence and argumentation used by stakeholders in the field, and; changes in institutional settings and priorities over time.

To develop out implementation history, we then took these emerging understandings of the PCNs’ ecology into the field, seeking to test our analysis of the material and capture the social factors outside the policies that have contributed to the longevity, popularity, and evolution of these particular PHC transformation efforts. We conducted 12 in-depth telephone interviews with 11 participants from February to April 2019. In these interviews we explored how participants perceived PHC transformation in Alberta generally, and the formation and evolution of the PCNs specifically. We used an interview guide that consisted of 10 open-ended questions and was developed, piloted, iterated, and then deployed to conduct the study (see supplementary file).

We employed purposeful and “chain referral” [45] sampling strategies to identify our research participants and policy documents. We developed an initial list of organizations and actors engaged with primary care transformation in Alberta through policy document analysis and a pilot interview. The key organizations identified through these sources were PCNs, the AMA, the MoH, AHS, and the Health Quality Council of Alberta (HQCA). In total, we interviewed 11 individuals of whom four were female and the rest were male. We interviewed clinical and administrative leaders from PCNs as well as PHC-transformation focused policy/decision makers from the organizations listed above. Our initial interviewees helped us identify other key actors (see list of interviewees per each organization in Table 1 below). We continued interviews until we reached saturation and no new observations on the nature and form of the PCNs’ implementation history emerged.

**Table 1:** Research participants with organizational affiliation

Organization	No
Primary Care Networks (PCNs)	4
Alberta Health (AH)	2
Alberta Health Services (AHS)	3
Alberta Medical Association (AMA)	1
Health Quality Council of Alberta (HQCA)	1
Total	11

The interviews were conducted or co-conducted by ML, AKM, and RPG, and ranged between 60 and 90 min. ML is a PhD qualified male tenure-track health services researcher with no medical training. AKM is a PhD qualified female health services research assistant. RPG is an MA qualified female health services research assistant. All interviews were recorded digitally and transcribed verbatim. Our research was approved by the Conjoint Faculties Research Ethics Board at the University of Calgary. We obtained, in advance of the interviews, written informed consent both to participate, and to allow recording from all participants. Transcripts were stored and analyzed securely with the assistance of NVIVO analytic software.

### Data analysis

We used an inductive approach [46] to analyzing both the documentary and interview data, drawing them into themes using content analysis methods [47]. Three members of the research team (ML, AKM, and RG) read policy documents and transcripts multiple times to identify emergent themes prior to engaging with stakeholders to check or expand on these initial understandings. For the interview data, we developed a preliminary codebook deductively based on the pilot interview and our previous analysis of the policy texts. We then expanded our coding inductively based on the themes and concepts that emerged from the data. We refined codes through constant comparison until a hierarchy of conceptual codes, and sub-codes emerged. We read, sorted, and re-read raw data repeatedly as categories and patterns emerged. An iterative process of challenges and discussions amongst the authors helped clarify distinctions between the categories and patterns. This iterative process helped decide the interpretations that most accurately portrayed our data [47]. Once no new concepts emerged, we finalized the coding framework. Data were coded using NVivo 12 software which enabled us to import and code the verbatim transcripts; review and re-code the data; and search for, and identify, categories and patterns [48]. The findings were also summarized, assessed, elaborated, and clarified in participant checks with our participants. Throughout the results section below, we use codes to identify these participants (P1 through P11). Any quoted transcripts have been redacted to remove identifying details, with changes noted in brackets, to protect the anonymity of individual participants and their organizations. As a further effort to protect anonymity, we have randomized the gender ascribed to our participants in the results section.

## Results

### History and evolution of PCNs

Our documentary analysis, interviews, and participant checked interpretations revealed two distinct periods in the evolution of Alberta’s PCNs: *The Frontier Era* (2003–2012), and *The Era of Accountability* (2013-Present). We describe each of these periods, focusing on the interactions between people, time, and culture as they shaped the implementation of the PCNs.

### The frontier era (2003–2012)

This initial era was characterized by a number of features: 1) the grass roots nature of the priorities and policies that the PCNs adopted; 2) clashes between the values and norms of the province’s powerful acute care system and the relatively uncoordinated primary care sector; 3) the universality of a problem for which local and highly variable solutions were sought; 4) the selective and limited use of evidence to build and measure progress for the PCNs, and; 5) the level of autonomy and independence afforded to the PCNs by AHS and the MoH.

In 2003, Alberta received $100 million of the Primary Health Care Transition Fund with the province opting to follow the five objectives the federal government had established at the time the fund was formed [28]. In this way, the LPCI program emerged and the three parties – the AMA, the MoH, and AHS – began fielding ideas, and designing programs. While there were conversations amongst all three, the AMA broadly, and a few committed individuals within the general practice section of the AMA specifically, took the lead. As one policy-maker described it:

[One family doctor] and his group of senior thinkers … said, “We’re up to the challenge, but you gotta help us. You gotta help us create a playing field where [the RHAs / AHS] will work with the PCNs rather than try to consume us. And you gotta help us with the other health professions so that we deliver integrated care through the PCNs rather than fragmented care.” [A politician] … had already started some fairly detailed discussions with [that same family doctor]. **(P1)**

The family physician’s concern that AHS would consume the nascent PCNs was one grounded in the functional, financial and political realities of Alberta’s healthcare system. While mandated with the organization and delivery of all healthcare in the province, the regional health authorities and eventually AHS, were and are heavily focused on the acute care system. As another of our key stakeholders noted, while primary care may be the preferred policy locus for broader PHC transformation, it is peripheral to existing healthcare operations in Alberta. He described how AHS has a relatively small group of staff focused on primary care,

I’m going to say about 100 strong within our large organization of [110,000 employees] … We’re a little bit like that glue that helps tie the [primary and acute care systems] together. **(P4)**

He went on to note how, in addition to primary care being peripheral to the core work of AHS, there was also the issue of culture:

What we’ve got here are two different cultures … operating from two completely different perspectives. And … the two aren’t even talking the same language, often. (**P4**)

A key cultural or linguistic difference to be bridged here is centered in the family physicians’ professional and vocational identities. On the one hand, the province’s legal and payment structures mark them as ‘independent contractors’ who provide care, hire and pay their own staff, purchase their own space, and bill the MoH on a FFS basis. And on the other, the broad scope of their work combined with their sustained, one-on-one relationships with their patients mark them as lone, long-term generalists. With both of these identities emphasizing longitudinal entrepreneurial self-reliance, it is perhaps unsurprising that there is a perceived gap with AHS’ acute, specialized, bureaucratic, system-reliant culture and language. Understanding this gap in the thinking and values of the two parties who enter into the joint venture that creates a PCN is essential to understanding the original formation of these organizations.

At a time of significant upheaval for the RHAs – they were in the throes of amalgamating into AHS – a small group of independent, lone, long-term generalists working in a field technically and culturally peripheral to what was seen as core healthcare operations, began talking to one another and directly to politicians. Out of these LPCI conversations came a vision, and the technical details of the PCNs. As one policy-maker noted, the vision coalesced

around local solutions for local problems. The local PCN basically got the dollars … in order to meet the needs of the local community. **(P9)**

Another participant from AHS repeated the mantra and emphasized,

there has to be flexibility, because PCNs were created [to deliver] local solutions for local problems. **(P7)**

In this way the independent professional and vocational identities of family physicians guided the initial vision for the PCNs. The technical details were also worked out in a culturally concordant manner. A physician leader described the homey conditions under which the PCN concept was drafted:

it was so small-town … We would sit on some guy’s living room carpet – two of us, with one person from the AMA – starting to sort of draft this [idea]. You know, go through this process. That’s literally how it started. **(P6)**

Down on the carpet, alongside the physicians, was an economist from the AMA who, as another participant described it

looked very much at the economic literature – at the papers and the books that were being published at the time, saying, “Here’s what the economic evidence might suggest.” To the best of my knowledge, he didn’t dig in to the medical literature. **(P3)**

In this way, the initial structures for the PCNs tended to focus on finance and economic issues, with the PCNs’ potential to tap into the QI, health services, care management, and primary care literatures laying dormant. As one of the physicians on the carpet noted,

I had no idea, really. I didn’t have the [QI] vision at that time, at all. **(P6)**

Another policy-maker participant used an Albertan cultural trope to describe not just these originating discussions on people’s floors, but the early years of PCN operations:

It was the wild west. **(P3)**

Another participant echoed this frontier image, emphasizing a key feature of the PCNs’ operational environment in the early years.

There were no … strings attached … So the PCNs [existed in a] kind of wild west sort of landscape. **(P5)**

At a high level, the cultural trope expresses a western Canadian narrative generally – one focused on frontier town initiative taking and small scale communitarianism in an atmosphere of few or no central rules. At a lower level, closer to the ground from which the PCNs were emerging, the wild west image was also concordant with the independent professional and vocational identities of family physicians.

Emerging out of these layers of culture, the PCNs were, as the previous policy-maker noted,

… pretty much just launched and left … and not followed through in an organized way by government until very recently **(P5)**

Another policy-maker participant concurred, noting,

Until we [developed] the [new] governance structure in 2017, each individual PCN was pretty much on their own. **(P9)**

PCN leaders affirmed the extent to which independence was a feature of their inception.

In the early days, we were pretty much left to our own devices. **(P2)**

In my recollection, there wasn’t a lot of “You should do this. You shouldn’t do that.” **(P11)**

The result of this culturally concordant approach to was that, in a frontier environment with few rules, bottom up policy innovation became the norm.

It was truly a grassroots movement … Very quickly, we got to a place where there’s a bunch of us that recognized there’s a real opportunity here, and we have to saddle this horse and ride it properly. **(P6)**

As part of attempting to saddle the horse, there were initially calls for the creation of a few model PCNs, however, this approach did not survive the first years of innovation. As one participant described it,

There was a belief amongst some that Alberta would be able to identify [a] handful of Centres of Excellence … and everybody else would copy them” (**P11**)

Instead, as a policy-maker noted,

the first PCN went live in 2005. And then, over the course of just a few years, we ended up with like 21 of them or something. That wasn’t the intent. So instead of having a couple [of] stellar examples, what happened was a lot of spread of PCNs across the province. **(P9)**

In this way the independent frontier environment supported a focus on locally adapted solutions and produced a wide range of sizes and organizational forms in what would, by 2019, become 41 PCNs across the province.

This structural heterogeneity reflected significant variations in the way the PCNs interpreted and sought to make good on the five federal objectives – subsequently adopted by the province – of the Primary Health Care Transition Fund. Where those objectives stressed PHC issues of access, health and wellness promotion, interdisciplinary team-based care delivery, and health and social services integration, the local priority for many PCN member physicians was the care of complex chronic patients. As one participant described it, if you had asked physicians at the time the PCNs were forming up ‘what’s the biggest problem in your practice?’ their answer would focus on one group:

*It was the complex patients. The ones that take a lot of time, more than a standard fee-for-service window would allow – whatever that may be. Some doctors may allow 10 or 15 minutes, some want to triple book every five. So whatever their definition of a standard window was, those populations were [seen as] problematic …* (**P2**)

As such, in many cases,

The PCN money [came to be] viewed as a supplement to the compensation of the family physicians. And that a lot of the per capitation money was re-distributed among the family physicians. (**P1**)

The FFS system of remuneration of the day was, in the case of complex patients with chronic diseases, seen as failing physicians and so the PCN capitation funding came to be seen as

a way to pay family doctors for all those things that they did that they weren’t being paid for” (**P6**)

While some PCNs merely funneled capitation money to their members, others took a more active, systems oriented approach to the challenges of delivering care to complex, chronic patients. Again, the approaches

*varied quite dramatically. Some places set up chronic pain clinics that still live today. [Others] looked at cardiac rehab [services]* that *still exist today. [Some] have foot care clinics that still exist today … We [also] saw a lot of PCNs say, “There’s gotta be a better way to get patients into specialty services. Maybe we’ll set up a Navigation Service, or a Referral Service that will help ease that burden on the primary care clinic and help patients more successfully make that transition.”* ***(P3)***

In this way the mantra of ‘local solutions to local problems’ was functionally converted into one that sought local solutions to what was, essentially, a universal problem (i.e., complex chronic patients).

Their degree of success in solving this ‘universal problem’ through their wide range of approaches was not something that was measured. The diversity of locally driven emphases and organizational arrangements, combined with no formal measurement or accountability requirements, meant the effects of the various innovations were virtually unknown. As one participant noted, the PCNs were, in the early years, a

completely evidence-free zone*.*

[Various stakeholders] had feelings, and they cited the evidence that supported their feelings. So, the pro [PCN] faction cited [the results of successful PCNs] saying, “Look at the wonderful things these PCNs are doing!” The anti [PCN] faction cited some of the scams that were going on, and said, “These people are just ripping off the Treasury!”

Did anybody say, “Well, here’s the percentage that are doing good, and the percentage that are scamming?” No, there was no objective analysis. The only actual analysis of it was done by the Auditor General. **(P5)**

In 2012, Alberta’s Auditor General (AG) delivered a report directly questioning the value that the province was receiving for its ongoing investment in the PCNs. At that point the federal money from the Transition Fund had been exhausted and so the capitation payments were now coming from the provincial treasury. The AG’s report, coming as it did amid the drafting of a new province-wide strategy for PHC, was generally accepted by our participants as ushering in a new era for the PCNs. It signaled the end of the Frontier Era and the arrival of Accountability.

In sum, the first decade of the PCNs was one rich in locally driven innovations and poor in accountability. With the professional vocation of primary care, the FFS arrangements under which the province’s family doctors operate, and Alberta’s frontier culture all valorizing and incentivizing independence, a profusion of PCN forms, sizes, and approaches to improving quality emerged in the early years. Steadfastly local in their outlook, and concerned, on behalf of their memberships, at the acute care system’s intentions towards them, the PCNs focused their diverse energies on the universal challenge of caring for complex chronic patients. From funneling per capita money directly to physicians, to setting up new patient services, their solutions ranged significantly and their ability to track, formally, success or failure was virtually non-existent. Throughout all of this, physicians and bio-medical approaches to care remained firmly at the centre of what was, in its funding mandate, to be a shift towards the principals of PHC. Focused on improving the care provided by family physicians to complex chronic patients, the progress made during the Frontier Era focused predominantly on a single population rather than population health more broadly. Preventative medicine, integration with other elements of the health system, quality improvement, and the delivery of care by teams that include non-physicians – all hallmarks of PHC – were also latent (Table 2).

**Table 2:** Key moments in PCN history (2012-19)

Time	Initiative/Policy	Content
2012	AG Report	Revealed major weaknesses in the design and implementation of PCN accountability systems.
2013	Alberta PCN Evaluation Framework	Developed by AH and AMA and set the foundation for Schedule B.
	PCN Evolution Vision and Framework	Used the term Patient Medical Home (PMH) for the first time.
	First Provincial Primary Health Care Strategy	Established five key strategic directions for continuing the transformation of primary care and its sustainability: 1) enhancing the delivery of care, 2) bringing about cultural change, 3) establishing building blocks of change, 4) population health, and 5) return on investment.
2016	Replacing 5 original objectives of PCNs by AH	Four new objectives include: 1) accountable and effective governance, 2) health needs of community and population, 3) patient medical home, 4) strong partnerships and transition of care.
2017	New Governance Structure	Consists of 5 PCN Zone Councils- forums for PCNs and AHS to collaborate in joint planning- and a PCN Provincial Committee

### The era of accountability (2013–2019)

The current era of PCN evolution has seen the continuation and evolution of features from the Frontier Era. Specifically, autonomy and culture clashes have persisted and come to be addressed through new mechanisms and structures of accountability. In addition, the present era has developed policy implementation characteristics of its own including the introduction of 1) PCN-level performance indicators; 2) governance and co-planning structures, and 3) Quality Improvement (QI) as a goal. We elaborate on these new features, noting developments and continuations.

### The arrival of accountability

The Frontier Era was officially ushered out by the AG’s 2012 report, although it was, arguably, already in motion as the MoH had been taking greater control over the form, if not the development and size, of the PCNs since 2008. As one policy maker speaking in 2019 noted, the problem of accountability was a foundational one.

The lack of clarity in exactly what those performance accountability requirements are … has been the challenge for the last 12 years. **(P7)**

This was a sentiment echoed by at least some of the PCNs for whom evidence of their successes and shortcomings was an important missing element. In the words of one PCN leader,

The accountability part is a question we’ve been asking since our very first business plan in 2004 – about how do we know we’ve made a difference. **(P2)**

The challenge, from the clinical and PCN perspective, being that performance measurement in primary care is confounded by the chronic, longitudinal nature of the work. The same participant noted:

We envy the acute care people because they can say “Oh, here’s a fracture. We fixed the fracture.” And six weeks later, the person is walking just fine. It’s a very obvious result. Primary care, particularly with chronic disease is [more a case of] “If we do this [one intervention] well, [the patient] will be in better shape 30 years from now.” [That sort of approach] doesn’t have very much political cachet. And so, a lot of [our performance measurement] is activity-based [showing] we’ve supported a lot of patients. **(P2)**

Another participant described the choice as one between following indicators of process or indicators of outcome.

You know, do we continue with process measures, or do we really just focus on outcomes and [the PCNs] figure out a way to get to those outcomes. **(P10)**

In this fraught, uncertain, and politicized measurement context, the AG’s 2012 report urged the MoH to, among other actions, establish clear expectations and targets for the PCNs, and to design systems for evaluating and monitoring their performance. Responding, the MoH worked with the AMA to create the PCN Evaluation Framework which would eventually lead to a set of performance indicators known as Schedule B.

As a policy-maker described it, the process of creating the Schedule B indicators was one of trying to make the highly localized operations of the PCNs not just amenable to measurement, but fungible across the system.

In order to get performance data out, you have to sort of do a lot of work around standardizing the [various] numerators and denominators so [the PCNs] can set those up in their systems. [To support] Schedule B – which is part of [the PCNs’] grant agreement – we have [measurement] toolkits. [Without the kits] what they report on isn’t apples to apples. [Developing those standards is] a whole process that needs to be ongoing. You can't do it all at once. **(P9)**

Schedule B’s measures of PCN performance focus on operationalizing 7 distinct policy goals through 9 indicators:

Developed out of the policy direction provided in the province’s 2014 Primary Health Care Strategy, Schedule B in this form was part of all the grant agreements between the MoH and the PCNs in 2019.

If Schedule B was, and is, a paper presence, the on-the-ground variations in PCN form, size, and service delivery mean there are gaps between the rule of measurement and its practical implementation. From the PCN’s perspective the developed standards remain open to significant local interpretation.

it’s a very subjective report in many respects. It doesn’t [tell a story like]“We made a million dollars and distributed the dividend to our shareholders.” I mean, it’s just not that precise. **(P2)**

With neither the work of primary care delivery, nor the indicators of Schedule B seen as amenable to the precision of corporate-style annual reports, MoH also finds itself in a gray area when it comes to enforcing accountability. As a policy-maker noted, because there has been

variability in the decision-making from the funder, [the PCNs] have a lack of clarity in exactly what some of those performance accountabilities and outcomes currently are …

If [a PCN] struggled and was unable to meet [performance targets] there was variability in the approach from [AH] as to how lenient or strict to be. So there’s some policy gaps there. **(P7)**

In sum, accountability has been, and remains, a key point of contention and effort for both the PCNs and the MoH. Gaining momentum with the 2012 AG’s report, AH’s efforts to gain a view of, and exert more control over, the PCNs, have been ongoing. Although driven by policy, the choice of indicators has proved a challenge to implement and enforce in the primary care environment. Nonetheless, the work of developing standards and then establishing reasonable tolerances for acceptable performance within those standards has emerged as a common project that the PCNs too have embraced.

### The arrival of governance and integration

**Fig. 2:**
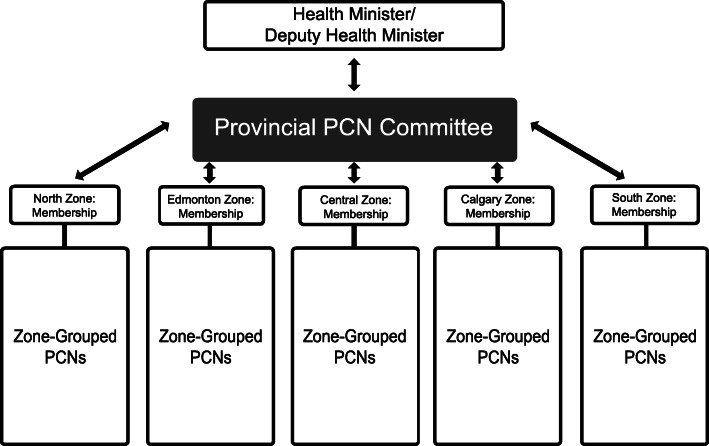
PCN governance structure

The move towards oversight through accountability was accompanied by a novel governance structure (see Fig. 2) that had, at least in part, its origins in what might be mistaken for Frontier Era policy generation. A physician leader described generating a rough draft of what would become the governance structure.

on a paper napkin for the deputy minister [of AH] in a cafeteria [and then turning it] into something concrete and then, socializing [it] and getting it ratified and voted on and accepted. **(P6)**

Sold as being a combination of integrative co-planning and a representative voice locally and provincially, the governance structure that was once merely a paper napkin attained more than 80% support from the 3800 family physician members of the PCNs in 2017. It created a structure in which AHS and the PCNs would co-plan their service delivery. The new governance structure includes a Provincial level PCN Committee - providing governance, leadership and strategic priorities - and five Zone level PCN Committees. The MoH, AHS, PCN physician leads, and a non-voting AMA representative are members of the Provincial PCN Committee. The sub committees are positioned at the AHS zone level, and so representatives from PCNs within each of the 5 AHS zones are now regularly drawn together with AHS personnel and community members to plan.

For their part, the cross-cultural meetings of Zone PCN Service Planning committees that bring together primary care and health system personnel, are, in their infancy. As one participant noted:

They’re just in the initial phases right now of starting to look at [co-planning]. And [the zonal committees are] looking at it from the perspective of some very specific populations. A lot of them are focusing on addictions and mental health: no surprise [there]. But another group is [focused on] the complex, elderly. Those kinds of things. **(P4)**

As this implies, while the policy direction for the zonal committees splits their focus across five populations – Well and At Risk; Maternal; Chronic Co-Morbid; Addiction and Mental Health; and Frail Seniors – the on-the-ground reality is one of locally chosen emphasis within these broad categories of policy and public health concern. In this way we see the search for local solutions to universal problems adapting to include consideration, and prioritization, of issues that have recently come to be seen, in Alberta, as major social and healthcare challenges.

As fledgling efforts at co-planning specifically, and trust and integration more generally, these zonal committees remain objects of concern for both sides. On the AHS side, one participant noted:

I’m not convinced that [the planning committees are] the great leveller. But I think there’s lots of opportunity for growth on both sides. Growth and learning. **(P4)**

From the other side, a PCN leader noted,

AHS feels an ownership of everything. They have a big ego, and it’s misapplied. Oftentimes I think of them as the kid at the candy store with their nose pressed up against the glass … It’s a candy store in primary care, and AHS wants to be part of it. **(P2)**

Another PCN leader used the same metaphor, describing how,

As the governance structure started to change, [our PCN’s] physician leader said to me, “You know, it’s like AHS is standing outside the candy shop scratching on the window, trying to figure out a way to get in.” **(P11)**

In describing what they see as AHS’s imperial ambitions, both participants are expressing the same anxieties that were present as the PCNs were originally created. Beyond reformulating a fear that the bigger organization might consume them, the PCN personnel are highlighting the size of the task that has been given to the local committees and the provincial-level body that oversees them.

In addition to creating lines of communication and accountability, the hope is that, through their work, the committees will mitigate the mutual mistrust of the two joint venture partners by bridging their cultural differences. These cultural differences are not just a theoretical challenge, but also a source of significant tension in the applied work of co-planning. As one participant noted, the PCNs find themselves caught up in their members’ cultural expectations and operational norms:

Primary care doctors are used to getting the information [from a patient] and making decisions and getting out of the [exam] room in 12 minutes. And they bring that same attitude towards their PCNs and their PCN operations. Now obviously, PCNs can’t make changes that fast. And so, [the doctors] already find PCNs slow. Then, [in the case of] PCNs working with AHS, AHS seems to move, from a primary care doctor’s [perspective at] a glacial speed. **(P3)**

Beyond frustration at the speed with which the larger bureaucracy of AHS moves, the PCNs and their members continue to assert their core cultural value: independence. What AHS or the MoH may see as policy directives to be implemented, the family care physicians see more as options on a menu. As a PCN leader noted,

the physicians on the front lines are kind of ‘a la carte accepting’ [of the various measures and priorities]. We are seeing good traction on the front lines for [some of] those. [But] it’s always a work in progress. It could always be better.” **(P3)**

Aware of the ‘a la carte acceptance’ engendered by the FFS remuneration structure and independence narratives of the family physicians, another participant highlighted the limits of AH’s capacity to push policy priorities downward. She posed the following questions:

How much accountability does the network membership – so the physicians and the clinics and teams – have to the actual PCN itself? The big challenge [has been] how do you get the adoption of these core priorities. How do you get the primary care docs to buy in and align their own practice priorities and plans [with the policy goals] so that you get a cascading upward effect? **(P10)**

While these questions have yet to be answered, the acknowledgement that alignment with PHC policy comes mostly from the bottom up rather than entirely from the top down, has been central to whatever success the zonal co-planning committees have enjoyed. A policy maker described how some of the committees have managed to avoid duplication, and so presumably save costs, while determining priorities from the bottom up:

A brand new program may come into place that AHS is creating, owns, and operates. And it could potentially be duplication of … some existing program that had been in a PCN business plan or [was already a] service plan priority. **(P7)**

The policy maker went on to note that it is “mostly AHS” that needs to amend and adapt its programming to avoid duplicating the work of the PCNs. This observation suggests both the relative nimbleness and responsiveness of the smaller organizations as well as their intimate knowledge of the populations they are serving as they make a la carte choices from policy priorities.

In sum, a governance structure at once desired by AH and popular with the PCNs’ family physician members arrived as part of the Era of Accountability. At the provincial level this structure provides the doctors with a voice upward, while acting as a downward conduit for AH priorities and thinking. At the AHS zonal level it is becoming a site for more or less fraught collaborations between the PCNs and AHS. In this co-planning space the cultural values and operational differences of the two joint venture partners are very much on display. Indeed, jealousies and perceived incompatibilities are just as likely as trust and consensus to characterize these early exchanges as relationships are being built. Given how new this cultural contact through substantive activity is, this is perhaps unsurprising. In any case, the zonal committees are facilitating conversations that would likely not have occurred in the Frontier era, or if they had, would have occurred in the isolation of individual PCNs with little chance of spread, scale or the emergence of standards at zonal or even provincial levels. Occurring in the Era of Accountability, these co-planning focused conversations are substantively focused on fostering greater mutual knowledge of one another’s activities with a corresponding reduction in duplicative programming. There is at least some perception that these gains are asymmetrical – with AHS seen as learning more about the wide variety of PCN programming that is already in the field, than the PCNs are learning about what they see as their AHS colleagues’ top-down policy and public health driven work. Beyond these perceptions and the substantive mission of the committees lies the hard and slow cultural work of developing good will towards one another.

### The arrival of quality

If the province’s 2014 Primary Care Strategy supplied the policy criteria for the arrival of accountability and Schedule B, 2 years later AH defined four new objectives for PCNs that have become, as the various players interpret and operationalize them, a focus on quality. The Auditor General report (2017) suggested that these 4 new objectives were aligned and consistent with the original 5 federal objectives that had launched the Frontier Era. In this way,

1. Accountable and Effective Governance;
2. the Health Needs of Community and Population;
3. the Patient Medical Home (PMH); and
4. Strong Partnerships and Transitions of Care

became the new menu from which PCNs and their member physicians began, and are now, making their ‘a la carte’ choices.

While policy makers and AHS personnel tend to see the governance, population health, and integration priorities on this list as equally important, their counterparts in the clinical and PCN world tend to emphasize the PMH. Indeed, the core mission of the PCNs in the view of rank and file as well as leader members, has in many ways, come to be seen as offering support for operationalizing the PMH. As one participant described it, the PCNs have,

a central, co-ordinating … administrative and support function [for the PMH] that is operationalized [in ways that are] somewhat up to the [PCNs] themselves, based on the needs of their members. **(P10)**

Another participant further underscored the PCNs’ presumptive role in supporting the changes required to enact the PMH. She noted that a large body of evidence

demonstrates that the majority of [family medicine] practices cannot [transform towards the PMH] on their own. A few early adopters can. But the majority of practices cannot. They require extensive coaching to learn*:*

How to be a team-based practice;

How to take best advantage of the resources that PCNs can bring;

How to manage their panel – now that they have panels, mostly;

How to do pro-active chronic disease management;

How to enact the medical home model. **(P5)**

In enumerating a number of the key features of the PMH – at least as they are stated in a recent iteration that can be traced from the original ‘patient centered medical home’ concept in the US, through versions espoused by the Canadian College of Family Physicians, to the AMA’s Towards Optimized Practice program – the participant provides a theoretical and technical understanding that is not necessarily front-of-mind for those on the clinical front lines. Another participant described how the ‘majority of practices’ in the PCNs experienced the arrival of the PMH as a single priority rather than a differentiated list of key features. He described how, as the PMH became part of the policy conversation, family physicians quickly cut to the chase asking:

“You want us to do quality improvement? Fantastic! So who’s gonna do that? Am I gonna do it, or do I need a person to do that? [A person] to help me to run my panels and my disease registries, and my screening lists, et cetera. **(P6)**

For the participant, as with many of the province’s operationally focused independent contractor family physicians, the PMH becomes synonymous with QI not so much as an aspirational mindset, but rather a set of tasks that will need to be carried out and paid for.

Those tasks might involve, at the low end of the spectrum, members seeking support in achieving the pre-requisite switch from paper to an electronic medical record (EMR). Further along the spectrum, it might involve support for understanding and deploying an EMR to work with now-empaneled patients to conduct population health interventions and improve integration with specialists and services in the community.

As one participant described it:

On the physician side, I think that there is a spectrum where there are a lot of physicians on the front lines who don’t know much about the PMH. But [others] in leadership positions have really embraced it, and are really helping to drive towards that direction. **(P3)**

Another participant described how, for him, Albertan

primary care is in its infancy in quality improvement. The new graduates coming out [of medical school] have some quality understanding and [are able to] apply it. But the vast majority of practitioners are really on the tip of what they need to be doing, or need to understand, from a quality perspective … Once you get quality improvement thinking, it changes the language, it changes the understanding [and it] opens the door to applied research evaluation. **(P4)**

As the participant elides the specificities of the PMH into a general capacity for QI, he highlights the idea that, setting the leaders and early adopters aside, the majority of family medicine practices need material assistance in understanding and applying the new principles of what they see as the preeminent policy option on the government’s menu. Material assistance here is not limited to finding ‘a person to do that,’ although this is clearly a necessary pre-requisite. In addition to providing incentives and supports, there is a need for improved front line understandings of the PMH/QI so that the measurement and evaluation expected by government are meaningful rather than tick-box exercises.

Where the physicians’ see the PMH as a menu of technical QI options within a menu of policy options, those in policy circles interacting with broader healthcare constituencies, see the ‘medical home’ as limiting and politically inappropriate. As a policy maker described it to an interviewer who had asked about the PMH,

You used the words “Medical Home.” If you said that to a room full of nurses, you might not survive. Cause there’s also the concept of a “Health Home.” And [non physicians] get very twitchy when you talk about medical home, because medical means “Doctor” in Alberta. And that means that you’ve already decided the primary caregiver is always going to be a physician. **(P1)**

This decision to have the primary caregiver be a physician was one that has been challenged as recently as 2012, the year that the AG’s report ushered in the Era of Accountability. As a physician leader described it, there was

at the time, a huge move from alternate providers, specifically Nurse Practitioners. They found favour with the premier [of the province]. And they were seen as somehow the new knights in shining armor that could somehow rescue primary care. And so, unfortunately, it became a turf war in the background between family docs and nurse practitioners **(P6)**

The move the participant describes was supported by the newly installed government of the day and sought to create Family Care Clinics (FCCs) run by Nurse Practitioners. The FCCs were, in the eyes of another physician leader, a politicized and ill-conceived attempt to resuscitate a concept that had proved unworkable in the US years before. He noted

Anybody who had a lick of sense could see … that the FCCs were unworkable. But [the government] were, nonetheless, pushing ahead with it. The PCNs were – and the medical profession were – screaming “Foul!” And the AMA was quite opposed to the FCCs as well. During that era three FCCs actually got launched. A few others were planned. I think one or two of the planned ones eventually happened as well. The whole Wave Two was abandoned. Wave Three was written off completely. As soon as [the government of the day was] replaced … the FCCs were just abandoned. **(P5)**

Having survived this bid from alternate providers and a hostile government it is perhaps unsurprising that PCN members are focused on the specifics of the PMH interpreted as QI.

Beyond being a casualty of a turf war, the health home concept does not, as a physician policy-maker noted, lend itself to winning the minds of that majority of independent family physicians who want to base their practice on evidence. He described the problem as one of credibility and authority,

The label – which is in the [academic] literature – is ‘Patient’s Medical Home.’ And it is really hard for us to change the label, and still be evidence-based*.*

I think we’d have gotten much further with the MoH if we’d used their preferred label, which is health home. [But], we can’t say to physicians, “You should move towards the health home,” and then point off at medical home literature without them going, “Well, wait a minute. Which one is it? We’re following the literature, not just [some] kind of random idea.” **(P3)**

The PMH, then, is both the intellectual grounding for a broader, *a la carte*, move towards QI thinking and activity, as well as an ongoing source of authority in a presently cold turf war with the allied health professions.

## Discussion

The PCNs’ ability to survive and expand not just locally, but across an entire province in what has been described as a ‘nation of pilot projects’ [49] which seldom manage to achieve spread or scale [50], is remarkable. Their key role in the province’s response to the COVID-19 crisis – distributing personal protective equipment and patient handling protocols to family doctors – indicates their ongoing importance. The cross-sectional implementation history we have presented here is limited to a moment in time, and relies on the insider perspectives of a limited number of key participants. As such, we have captured a single phase in the PCNs’ evolution, and may have missed important perspectives outside their original generative group. With calls now being made for the PCNs to become *the* engines of quality and transformation at a time when alternatives to FFS are being considered [40], the account we offer is undoubtedly relevant in policy circles. Where the data to support this future QI work, or to provide accountability and planning capacity for the MoH, were initially absent, these measures have begun to come online. That said, the operationalization of Schedule B (See Table 3) has been and remains highly variable. This is true both as PCNs wrestle with measuring a lack of illness and diverted or prevented contacts with the acute care system, and the MoH varies in the financial penalties it exacts for missing indicators. As the parties interact and ways of implementing the measures take a consistent hold, the PCNs have managed to balance and rebalance the priorities of, on the one hand, their member physicians, and on the other the provincial payer. The key factors facilitating this evolving capacity to balance, and so achieve longevity, popularity amongst physicians, and steps towards PHC transformation, can be organized into the following categories: people, time, and cultural adaptation.

**Table 3:** ‘Schedule B’ Measures of PCN Performance (source: Auditor General Report, 2017)

PHC System Outcome	PCN Level Performance Indicator
**Attachment** All Albertans have a ‘health home’	1. Percentage of patients going to a different provider or different clinic for a subsequent visit.
**Access** Albertans have timely access to a primary health care team.	2. Percentage of physicians measuring Time to Third Next Available Appointment (progress measure for actual mean time to TNA).
**Quality** Clinical and social supports are brought together to promote wellness, provide quality care based on proven courses of action, and effectively manage chronic disease.	3. Average of patient responses to the question “Overall, how would you rate the care you received in your visit today?”
	4. Percentage of compliance of physicians in screening or offering screening to their panel of patients, as described in a menu of screens recommended by Alberta Screening and Prevention Initiative (ASaP).
**Self-management of Care** Albertans are involved in their care and have the supports needed to improve and manage their health.	5. Percentage of patients with a chronic condition who were offered self-management supports during the fiscal year.
**Health Status and Care Experience** Albertans are as healthy as they can be, have better health overall, and report positive experiences with primary health care.	6. Percentage of patients with a chronic condition who report maintaining or improving quality of life as measured by the EQ-5D Health Questionnaire during the fiscal year.
**Provider Engagement and Satisfaction** Providers satisfied and happy with their work lives and able to provide quality care.	7. Percentage of identified team members responding to a team effectiveness survey.
**Leadership and Governance** PCN leadership and governance is effective.	8. PCN board completion of all three components of self-assessment during the fiscal year: • self-assessment of the PCN board as a whole • self-assessment of individual PCN board members • performance improvement plan
	9. PCN board assessment of the performance of the PCN administrative lead and all other staff members reporting directly to the board for the prior fiscal year.

### People

As the first of the province’s 41 PCNs began their existence on a living room floor, a small group of people took advantage of the flexible starting conditions and began to operationalize the Primary Health Care Transition Fund along with its five policy goals. Their success in creating organizations to bridge the gap between the payer and the front lines of primary care was something the literature suggests was far from a foregone conclusion. Indeed, it rested on the small group’s ability as ‘policy entrepreneurs’ [51–53] not just to formulate a viable program, but to read the political landscape, work their abundant and high level connections to government and physician colleagues, and to socialize their ideas more broadly. These entrepreneurs provided not just a practical form but a moral core to the PHC message. Practically, they designed the first organizational structures, and indeed subsequent governance reforms, in ways that would both protect the emerging PCNs from being consumed by the significantly larger acute care system, and engender the trust and support of rank-and-file family physicians. In this sense the policy entrepreneurs more or less consciously designed social acceptability, and so legitimacy [54], into the PCNs from the beginning. Later, as the Era of Accountability arrived, they were joined by others from clinical and policy-making backgrounds to continue this process. The now enlarged cohort of entrepreneurs, designed in not just the ability to develop local solutions to a universal problem, but a voice for family physicians in how the system generally, and the transformation towards PHC specifically, would occur.

These elected Physician Executive Leads from each of the zones now speak to, and on behalf of, the province’s more than 4000 PCN members, in a governance structure that is unique in Canada. Through the new governance structures – the provincial level, and zonal level committees – this expanded group created, socialized, and ratified spaces of cultural interaction that were both bi-directional communication links with the MoH and AHS, as well as a presence for primary care as a distinct specialty in an AMA environment previously dominated by acute and other specialist care. Morally, the entrepreneurs have spoken consistently of improving care, more or less following the script of PHC transformation by adopting the PMH. Without this message of care quality improvement as a central mission, the PCNs ran the very real risk of becoming mere money conduits – funneling capitation funds to individual physicians as a salary top up – and so being shut down as the AG ushered in the Era of Accountability. The choice to pursue the PMH as a substantive program for interpreting and achieving PHC is telling in that it, along with much of the data we have presented here, is focused on physician activity, and bio-medical understandings. This is to say, the PCNs’ popularity and longevity cannot be attributed exclusively to their QI mission or PHC commitments. Rather, they hold their family physician-members loyalty in at least some measure because they unabashedly assert the centrality of medicine and medical expertise in the delivery of PHC’s population health, prevention, team-based care, and QI goals.

Similarly, the rise of PCNs has been contemporaneous with family physicians acquiring a voice and place in a provincial system which previously had little time or consideration for them. A major benefit of this strengthened position relative to hospitalists and specialists has been the capacity to provide a robust and coordinated response to professional encroachments [55] such as moves made by allied health practitioners and politicians seeking to set up FCCs. It is noteworthy that the small group of entrepreneurs, as central as they were to the formal creation and moral orientation of the PCNs, did not come to their work as fully formed experts in PHC transformation.

### Time

The original policy entrepreneurs at the practical and moral centre of the PCNs were not, initially at least, the sort of QI champions often described in the literature. Yes, they believed, or bought-in to [56], the broad idea of PHC transformation, but they began their work with neither a particular vision, nor expertise beyond their clinical experience, nor evidence-based examples of what they were about to implement. Rather the PCNs were built according to the economic literature. The transformative vision, as well as the at-the-time nascent tools of QI, would take time to develop as the core group of people expanded and ideas arrived from academic and policy environments. In this sense the original group began as policy entrepreneurs and became, in the context of the PCNs they built with the colleagues they could convince and assure, QI champions. It was time that allowed them to learn, experiment, and discover inside their flexible starting conditions [57, 58]. With this came not just knowledge and expertise but the ‘buy in’ conferred by construction and ownership [59–61].

Time has also, in combination with the committees and co-planning obligations of the newly created governance structures, made space for intellectual and cultural contact between the worlds of clinical, administrative, and policy people. In this way time and space have been central to overcoming mistrust and beginning the transformation towards PHC. For their part, the PCNs have benefited from the time required to move from feeling their way in a new space with few doctors signed up as members, to being confident, seasoned teams able to function not just in the Frontier Era of few rules and little oversight, but in an era of Accountability where they act as hosts for difficult cross-cultural conversations amongst the MoH, AHS, and the vast majority of the province’s family physicians who have signed up. The decade or more of sheltered time to build programs, tear down, and try again has been invaluable, allowing the PCNs not just to adapt their services to local conditions, but to transform themselves from being disbursers of capitation funding to players at the table, co-planning services with the larger acute care system.

### Cultural adaptation

Meeting what have been called the ‘adaptive challenges’ of change management generally [62] and healthcare quality improvement specifically [63–65] has been identified as a necessity when seeking to transform the practice of not just FFS family physicians in Canada, but around the world. Adaptive challenges here are those tied up in the values, social practices, policies and path dependencies – writ large, the culture – of the operational environment where transformation is being attempted. The implementation history of the PCNs suggests an evolving and nuanced role for cultural adaptation as a strategy for achieving longevity, popularity, and transformation towards a physician-focused interpretation of PHC. Our findings suggest a super structure of cultural transformation has emerged from and been made possible by a base layer of requisite, legally re-enforced cultural adaptation.

Where some health systems outside of Canada have what might be called command-and-control relationships with their family physicians, the MoH and AHS along with their counterparts in the other provinces work with independent contractors, not salaried employees. Furthermore, those contractors enjoy significant legally protected autonomy when it comes both to treating their patients, and in their relationships with the rest of the health system [30, 39]. In Alberta, as elsewhere in Canada [26], this independent contractor status, and the autonomy it affords physicians, are legacies of decisions made in the 1960s as Canada’s universal publicly funded healthcare system was put in place [26, 32]. These legal legacies are overlain by a western Canadian frontier narrative, as well as professional and operational cultures that valorize the ideal of lone generalists enjoying long term relationships with their patients [66, 67]. Given this admixture of law and culture it is perhaps unsurprising that these independent contractors, or ‘non-system’ members of the Canadian health system, have proved resistant to ‘system’ changes [26, 30]. While Alberta and Canada will be idiosyncratic in some ways, this clash between the cultures of physician and system is a phenomena observed in jurisdictions around the world, even those with more command-and-control capacity [68, 69]. Acknowledging this reality, policy makers aiming to achieve transformation towards PHC have focused on voluntary, discretionary, participatory, and incentivising mechanisms to secure the buy-in of ‘non-system’ members [70]. As such, opting for local (not central) solutions, and a fragmentary ‘a la carte’ (not a unitary, command-and-control) approach to PHC policy implementation was, in many respects, a necessary foundation on which to build the first PCNs.

A culturally concordant, as well as economically viable, way of doing the business of PHC was at the core of how the original policy entrepreneurs designed the PCNs. They emerged from the mutually reinforcing values of the frontier town thinking –Canada’s particular version of the Wild West cultural trope – economic entrepreneurialism, and primary care’s identities and values [71]. These three tropes overlap at, and emphasize, self-reliance, independence, autonomy, and rapid locally initiated, locally tailored action [72]. As identified elsewhere, acknowledging and adapting interventions to these closely held cultural values is clearly a key element in making PHC transformation not just possible [68] but seen as legitimate by family physicians [73]. In Alberta’s case that legitimacy, generated out of cultural concordance and a sense of local ownership of self-made solutions has held [61, 74, 75]. Indeed, it has thrived, with over 80% of the province’s physicians making the decision to sign contracts and remain members of their PCNs even as the Era of Accountability with its opposing cultural values arrived. The PCNs have survived and grown in popularity in part because they offer a cultural redoubt where independence and local action are protected and respected.

Their culture and that of their fiercely independent physician members, has been transformed so that accounting to achieve the PMH, and concern for broader system narratives have become part of the conversation and moral calculus. Acceptance of the system’s values and methods in the form of policy packages focused on transforming towards PHC and spreading QI thinking, have become part of what the PCNs do. Performance metrics – arguably the basis for so much of the QI work that needs to be done – have asserted themselves at the PCN level, introducing the potential that they might touch down with individual physicians. While these might be seen as ‘victories’ of a sort for system culture, this sort of zero-sum approach would require us to tot up points for a physician culture apparently in retreat. In doing so we would see that PHC transformation activities are presently interpreted through the lenses of what are now 41 different, member-driven PCNs. On the one hand, this has led to a proliferation of local solutions to universal problems which are robust and fit for purpose. And on the other it has led to ‘a la carte’ choice making from what family physicians tend to see as a menu of policy options, but others in the system tend to see as fixed groupings. Setting aside a zero-sum account of cultural victories and defeats, it is perhaps more appropriate to note that the two cultures, finally in real and meaningful contact with one another, have begun a transformative dialogue.

Underpinning the success of this dialogue have been people from both cultures who appear, more or less consciously, to have discovered common ground and indeed institutionalized it in the form of the PCNs and their governance model. The PCNs provide a contact point for the two discordant cultures, and out of this contact they have found shared value: flexibility. Flexibility in interpretation and application of otherwise fast rules is grumbled about, but ultimately prized, by both the PCNs and their acute care and policy counterparts. Just as the PCNs choose which parts of the PMH to emphasize, our participants suggested the MoH also exercises discretion, shielding some PCNs who miss performance targets and holding others sharply to account. This, we argue, suggests that the people inside the PCN structures have, as part of transforming not just towards PHC, but their own cultures and understandings, prefer flexibility in how they operate. The value of this cultural feature – this rough agreement on the importance of flexibility – is that it appears to bridge the other divides that separate the players and so supports the transformation agenda [76]. Without the PCNs the common ground and conversation required to move, however incrementally, towards transformation would not exist. As much as the PCN reflect the small-scale communitarian values of family physicians they have also created a toehold for central accountability and QI. People on both sides of the cultural divide have worked, over time, to create performance metrics that are locally meaningful and facilitative of the broader transformative mission rather than merely seeking to exert control from above. The arrival of Accountability in the form of Schedule B measures, co-planning requirements, and QI as a central activity, confirms not just the importance of what have been called ‘hard edges’ [77, 78] in the difficult and slow work of transforming towards PHC, but of cultural adaptability. Efforts to support the positive outcomes of flexibility, and effectively corral the negative potential so that an entire province of family physicians rallies around a full PHC policy package, have been at the core of the PCNs’ evolution.

## Conclusions

Alberta’s experience highlights the potential organizations like the PCNs carry for accomplishing PHC transformation in a FFS, government payer environment. Leveraging the zeal of policy entrepreneurs and the time both to develop as organizations and to establish their capacity to benefit member physicians, the PCNs have begun to take on a greater role. Their story, and the success they are enjoying as instruments of physician-driven QI and accountability, hangs on their ability to facilitate conversation and walk a line between cultural values of independence and autonomy on the one hand, and accountability and quality control on the other. The broader lessons for those seeking to support PHC transformation in similar FFS environments are: Begin from a broad commitment to improving quality, but be prepared to proceed flexibly; Provide time for individuals and organizations to learn about quality improvement, one another, and how best to support the transformation of a system while delivering care locally; Create physical and policy spaces where cultural contact can occur as these are central to achieving ‘buy in’ and to easing mistrust.

## Supplementary Information

**Additional file 1.**

## Data Availability

Links to, or citations of, government documents used in the analysis are included in the reference list. The raw, and thus un-anonymized, interview transcripts from the study are not included as an appendix, or otherwise available for other researchers to consult. This is in keeping with best practices in the conduct of qualitative health services research, and the specific commitments to anonymity laid out in the ethics board approval for this study.

## Abbreviations

AHS: Alberta Health Services
AMA: Alberta Medical Association
FFS: Fee-for-Service
LPCI: Local Primary Care Initiatives
MoH: Ministry of Health (Alberta Health)
PCN: Primary Care Network
PHC: Primary Health Care
PMH: Patient Medical Home
QI: Quality Improvement
RHA: Regional Health Authority
