# Supplementary material for: An implementation history of primary health care transformation: Alberta’s primary care networks and the people, time and culture of change
Source: BMC Fam Pract. 2020 Dec 5;21:258. doi: 10.1186/s12875-020-01330-7 (PMC7718828; doi:10.1186/s12875-020-01330-7)
Supplement: Supplementary file 1 — Additional file 1. [file 12875_2020_1330_MOESM1_ESM.pdf]

Interview Guide

1. Please tell me about your position, how you came to it, and what it involves?

2. Tell me about the patient medical home (PMH).
   a. What does the PMH mean to you?
   b. What are its key concepts and components?
   c. If 'NO IDEA' answer:
      some of the key concepts and components that have been discussed in the literature are: *researcher to state 10 goals for Family Medicine Practices to pursue* - from The College of Family Physicians of Canada

3. In your opinion, is the PMH being delivered within PCNs?
   a. If 'YES' answer:
      - How so?
      - Are there practices and/or PCNs who are having more, less, or about the same level of success?
      - What, in your opinion are the factors making delivery of the PMH work?

      Probe on: PCN membership, personnel and team; policy; payment; patient mix; other?
   b. If 'NO' answer:
      - How not?
      - Are there practices and/or PCNs having more, less, or about the same level of success?
      - What, in your opinion are the factors that are making delivery of the PMH so difficult?

      Probe on: PCN membership, personnel and team; policy; payment; patient mix; other?

4. Performance metrics for primary care have become a major point of interest for policy-makers, citizen-patients, and health care providers alike. There seems to be agreement that measuring how primary care is doing, is a good idea, but there doesn't seem to be a lot of agreement on which parts of primary care ought to be measured. Is that a fair assessment?
   Why? Why not?

5. It seems like maybe health professionals are interested in metrics that can be used to evaluate PCN team performance and remuneration plans; and that citizen-patients are interested in PMH metrics that focus mainly on experiences of patient care. Does that sound reasonable?
   Why? Why not?

6. What is the most important use of PMH performance measures from your perspective as a policy-maker?

Ethics ID: REB18-0171

7. We have talked about factors that contribute to the successful delivery of the PMH. With this in mind, what elements of the PMH would you want to have measured to assess performance?

a. For 'NO IDEA' answer: researcher to identify several metrics (see Appendix H - Schedule B Primary Health Care Indicator Set) for a complete list of metrics.

b. For 'YES' answer follow up with:

- Are there any others? Are there any others you would **not** choose? Why?
- If you were to put on the hat of a health care provider or citizen-patient, are there metrics you would choose? Why?
- How do those choices resonate with you as a policy-maker? Is there a middle ground position to be found, where all three groups get what they are looking for out of the PMH performance measurement? Who has to give up what?

8. PMH performance measurement doesn't occur in a policy and practice vacuum. Are there other elements that would need to be changed to make performance measurement in primary care more possible and practical?

a. I'm thinking particularly here of changes to health care provider payment structures, or clinical governance models, but maybe there are other things in the policy environment that would need to change?

9. Is there anything we haven't talked about that you would like to discuss?

10. Would you be interested in participating in the de-briefing sessions in Phase II and/or the Consensus Conference in Phase III of this study?

a. If response is 'YES': "A member of the research team will contact you in approximately the next 12 months, using the contact information you have provided us with today."
